# E-Bicycle as a Green and Physically Active Mode of Transport from the Aspect of Students: TPB and Financial Incentives

**DOI:** 10.3390/ijerph20032495

**Published:** 2023-01-31

**Authors:** Nenad Djokic, Nikola Milicevic, Branimir Kalas, Ines Djokic, Vera Mirovic

**Affiliations:** 1 Department for Trade, Marketing and Logistics, Faculty of Economics in Subotica, University of Novi Sad, 24000 Subotica, Serbia; 2 Department for Financial and Bank Management, Faculty of Economics in Subotica, University of Novi Sad, 24000 Subotica, Serbia

**Keywords:** e-bicycle, physical activity, green transport, student population, theory of planned behavior, attitudes, subjective norms, perceived behavioral control, financial incentives

## Abstract

The positive effects of e-bikes on physical activity, health, and the environment have been confirmed in many studies. Their choice, as well as of cycling in general, was previously considered from, among others, the socio-psychological aspect (often by use of the theory of planned behavior (TPB)) or the financial aspect (in the context of financial incentives). In addition, the question of physical activity can be especially relevant for the student population, since their level of physical activity usually declines. Starting from the previous framework, the aim of this research was to consider the intention to use e-bikes by the student population in the context of their attitudes, subjective norms, perceived behavioral control, and financial incentives. It is, according to the authors’ knowledge, the first research that combines all those variables when studying e-bikes. The research was conducted in 2022 on a convenience sample of 332 students from the University of Novi Sad (Republic of Serbia). The results show that the strongest predictor of the intention to use e-bikes can be attributed to financial incentives, followed by attitudes and subjective norms, while perceived behavioral control is not significant. Besides considerations in the context of previous research, additional recommendations for increasing e-bikes’ use were provided.

## 1. Introduction

Physical activity is considered an important determinant of health in everyday life [1]. It has a significant role in people’s healthy functioning [2], as it provides many physical benefits, including body weight maintenance, better bone health, larger muscle strength, lower blood pressure, etc. [3]. In addition, physical activity provides psychological benefits, such as the improvement of cognition and brain function, and the reduction of depression and dementia risk [3]; it participates in the promotion of the mental state by maintaining positive emotions and an optimistic attitude [4]. Hence, a lack of physical activity may result in people becoming prone to a long list of diseases, including high blood pressure, type II diabetes, coronary heart disease, anxiety and depression, certain types of cancers, obesity, asthma, osteoporosis, dementia, etc. [2].

When it comes to physical inactivity, special emphasis should be placed on the student population. At these ages, the levels of physical activity usually decline—as everything is digitalized in today’s society, with students spending a lot of time sitting in front of their computers (laptops), TVs, and smartphones [2]. The decline in students’ physical activity may also occur due to changes in their social environment and interpersonal relations, lower parental control, less access to structured physical activity, and greater school and/or work obligations; the problem may also be of a motivational nature—when beliefs and emotions are not in accordance with desired physical activity outcomes [5]. The study by Grasdalsmoen et al. [6] conducted among Norwegian students showed that the large majority did not meet the international recommendations concerning exercise, whereby the prevalence of overweight increased in both genders. According to research from the Republic of Serbia, performed by Malčić and Marić Jurišin [7], the real moderate physical activity of students, as most productive for a healthy lifestyle, was below the recommended minimum.

In order to increase physical activity, and thus improve health, students, as well as other age groups, can turn to cycling. The development of technology has contributed to the emergence of different bicycle types, among which are electric ones. This segment of the transportation system is one of the fastest-growing [8]. Its global market worth in 2021 was estimated to be USD 35.29 billion, with the potential to reach more than USD 92 billion by 2029; in Europe, Germany has the largest e-bike market (44% of e-bike sales), followed by the Netherlands (21% of sales) [9]. Because of their potential benefits for both the riders and the environment, this research focuses on students’ intention to purchase e-bikes. For this purpose, we relied on the theory of planned behavior, i.e., its main predictors (attitudes, perceived behavior control, and subjective norms). Hereby, as e-bikes are much more expensive than conventional ones, in accordance with a similar study [10], financial incentives were also included as a predictor. After presenting some aspects related to electric bicycles, physical activity, TPB theory, and financial incentives, the conceptual model and hypotheses were introduced. To examine the relations between variables, structural equation modeling (SEM) was implemented. The obtained results are accompanied by a discussion and conclusion.

## 2. Literature Review

### 2.1. Electric Bicycles and Physical Activity

One of the first versions of an electric bicycle dates back to 1895, i.e., to the invention of Ogden Bolton; it was “an EB integrated with a six-pole direct current (DC) hub motor mounted in the rear wheel” [11] (p. 3). Since then, not only technology has changed, but also the understanding of the term electric bicycle. According to Hung and Lim [12], there are three main types of electric bicycles: the pure electric bicycle, whose driving power is based only on the electric motor, without any human power; the power assistant or electric assistant bicycle (EAB), also known as a “pedal electric cycle” (pedelec), in which the rider uses an electric motor as an assisting power when pedaling; and the electric bicycle with two modes, pure and power-assisted. Depending on the design, performance, and control mode, Fishman and Cherry [13] distinguished different variations of electric bicycles (e-bikes) from bicycle style to scooter style. Although in the literature both bicycle-style e-bikes (BSEBs) and scooter-style e-bikes (SSEBs) refer to the term “e-bike”, in North America, Europe, and Australia, e-bikes are usually related to BSEBs [13]. In the European Union, a very popular electric bike is a pedelec (or pedelec-25), with a motor whose power is limited to 250 W and whose assistance turns off at speeds over 25 km/h. Contrary to this type, all other e-bikes are subject to approval regulations and are classified into L1e-A “powered cycles” and L1e-B “mopeds” [14]. While the former relates to pedaling cycles with motors limited to a speed of 25 km/h and power of 1000 W, the latter refers to vehicles whose maximum speed is between 25 km/h and 45 km/h, and whose motor power is between 1000 W and 4000 W.

Pedelecs can bring many benefits to riders. With their use, people can travel longer distances, carry greater loads, and overcome obstacles such as slope and physical effort [15,16]. In addition to facilitating the transport itself, electric assistant bicycles have economic and environmental advantages. Compared to a car or a gasoline scooter, for which the total cost per km (including the purchase, energy consumption, and maintenance) amounts to USD 0.62/km and USD 0.031/km, respectively, in the case of an electric bicycle, it is less than 0.7 cents [17]. From an environmental perspective, the favorable impact of e-bikes reflects in a decrease in greenhouse gas emissions [18]. The use of an electric bicycle as an alternative transport mode can prevent pollution, bearing in mind that in urban areas the emissions of a petrol car include HC (hydrocarbons) of 3.57 g/km, CO of 3.15 g/km, CO_2_ of 1.82 g/km, and NOx of 2.29 g/km [17]. Considering lifecycle CO_2_ emission rates and travel displacing estimates induced by e-bike usage, it can be expected that each adoption of this transport mode will result in a reduction of CO_2_ net emissions exceeding 460 kg per year [19]. Moreover, as stated by Torregrosa Mira et al. [20], the environmental advantage of e-bicycles relates to the fact that they make almost no noise.

When it comes to electric bicycles, an important topic, which has been investigated in a number of studies, relates to physical activity and health. Berntsen et al. [21] compared e-biking to conventional bicycling, for which purpose two different routes were used; their results showed that although cycling on an e-bike was faster and less intensive, most of the time, both activities were on the MVPA (moderate- and vigorous-intensity physical activity) level. Therefore, in order to increase the level of physical activity, e-bikes should be used for longer distances, especially as an alternative to a car when commuting [21]. Castro et al. [22] compared the levels of physical activity between e-bikers and conventional cyclists, as well as among e-bikers depending on the transport mode substituted by an electric bike. Their findings suggested that when switching from conventional bicycles to electric ones, the net losses in physical activity were lower because of the overall increase in traveling distance; on the other hand, there was a substantial increase in physical activity when switching from public transport and private motorized vehicles to e-bikes. Following the research of Bourne et al. [23], which was based on 17 studies, there was moderate evidence that for both physically inactive and active persons, electrically assisted cycling provides moderate-intensity physical activity; in addition, moderate evidence was found in relation to the positive impact of e-cycling on cardiorespiratory fitness in the case of physically inactive persons. Anderson et al. [24] examined the effects of e-bike usage on health and well-being, taking into account inactive and overweight people. The analysis indicated that e-biking evoked feelings of happiness; it positively impacted physical and mental health and improved the overall sense of well-being. The positive effects of e-bikes on physical activity and health have been confirmed in many other studies, including those conducted in the Netherlands, Switzerland, the United States, and Germany [25].

### 2.2. Theory of Planned Behavior

As stated in the study by Conner and Armitage [26], “the theory of planned behavior is a widely applied expectancy–value model of attitude–behavior relationships” (p. 1429). The theory of planned behavior, as well as the theory of reasoned action (TRA), whose extension TPB presents [27], was developed in order to explain informational and motivational impacts on behavior [26]. The central factor in both theories (TPB and TRA) refers to a person’s intention, which in accordance with a general rule positively influences behavior; on the other hand, the main predictors of intention are attitudes (a person’s favorable or unfavorable evaluation of the behavior), subjective norms (perceived social pressure related to behavior performing), and perceived behavior control (perceived easiness or difficulty of realizing the behavior), whereby it is expected that all three predictors have positive relations with the intention [27].

The theory of planned behavior “has received considerable support in a large number of empirical investigations” [28] (p. 323) covering different research fields. It was used in studies related to biking, with a focus on cycling behavior [29], intention to use a bike [10,30,31], and bike sharing [32,33,34]. Besides conventional bicycles, the TPB theory or its factors were applied in studies related to electric bikes. Yasir et al. [35] used an extended theory of planned behavior to examine e-bike adoption intentions on a sample of Chinese bike riders. Among the others, all three TPB factors (attitudes toward e-bike adoption, perceived behavioral control, and subjective norms) significantly and positively affected intentions to adopt e-bikes. Simsekoglu and Klöckner [36] analyzed the impacts of several factors on the intention to use an electric bike in Norway. Among those factors, attitudes as a TPB predictor had a significant and positive effect on the intention to use an e-bike. The extended theory of planned behavior was applied by Li et al. [37] for examining the intention to use shared electric bicycles, whereby attitude, subjective norm, and perceived behavior control were found to be significant predictors that positively affected the analyzed intention.

### 2.3. Financial Incentives

Due to potential benefits related to greater physical activity in the population, and car replacement when traveling, many governments are interested in stimulating electric bicycle implementation [38]. Hereby, there are different types of incentives that can be used, such as monetary incentives to purchase a bike, free use of electric bicycles, vouchers, gifts, and monetary rewards based on smartphone applications [10].

In a number of papers, incentives have been analyzed in relation to bicycles, both conventional [10,39] and electric. Ciccone et al. [40] examined cycling activity from the aspect of different types of economic incentives, including participants who required access to a bicycle or electric bicycle; following their results, a flat rate, as well as a conditional lottery, positively influenced people to cycle more. A study by de Kruijf et al. [41] pointed to the effectiveness of the e-cycling incentive program in the province of North Brabant, the Netherlands, which was based on monetary incentives for using e-bikes; among others, they found satisfactory results as 66% of the commute trips were performed by electric bikes one month after the start of the program. The research of Sundfør and Fyhri [42], conducted in the context of a subvention program introduced by the Oslo City Council, indicated that financial incentives for purchasing e-bikes can lead to an increase in active transport, even in the case of a simple subvention that is not focused on specific population segments.

In Europe, many countries offer tax incentives and purchase premium schemes for cycling; in regard to pedelec-25, some incentives on a national level are presented in Table 1. They can be intended for different target groups (individuals, businesses/associations, and/or public entities).

In addition to the national authorities, incentives are offered at regional and local levels. For example, in Piemonte (Italian region), individuals could receive EUR 500 per pedelec-25, while in Bari (Italian city) that amount was EUR 250; they represent only a small part of almost 300 cycling schemes that are (or were) offered across Europe [43].

## 3. Conceptual Framework and Hypotheses Development

Following similar studies related to the analysis of customers’ intentions, this research is also based on the application of the theory of planned behavior. Therefore, the intention to purchase an electric bike was examined in relation to three main TPB predictors (attitudes, perceived behavior control, and subjective norms). Bearing in mind the assumption according to which the individual’s intention toward certain behavior will be stronger if subjective norms and attitudes are more favorable, and perceived behavior control is greater [27] (p. 188), we defined three hypotheses:

**H1:**  *Attitudes significantly and positively affect the intention to purchase electric bicycles.*

**H2:**  *Perceived behavioral control significantly and positively affects the intention to purchase electric bicycles.*

**H3:**  *Subjective norms significantly and positively affect the intention to purchase electric bicycles.*

**Figure 1:**
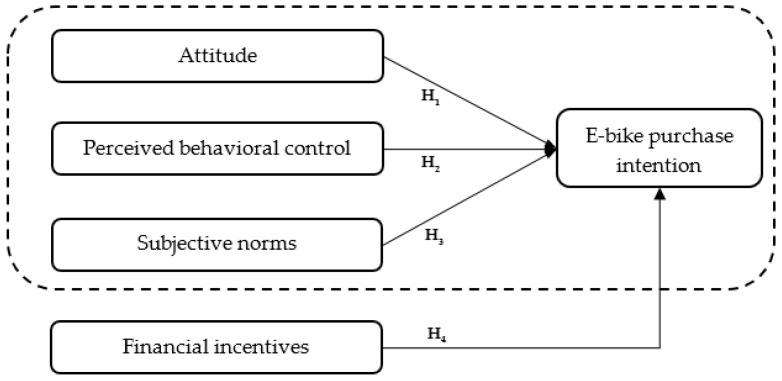
Conceptual model.

Besides TPB predictors, the model (Figure 1) included financial incentives as an antecedent of intention to purchase electric bicycles. The importance of this variable lies in its potential outcomes related to the use of e-bikes; moreover, research by Baeli et al. [10], which was also based on the application of the TPB framework, showed that financial incentives have a strong influence on the intention to use a bicycle. Therefore, the fourth hypothesis is:

**H4:**  *Financial incentives significantly and positively affect the intention to purchase electric bicycles.*

## 4. Materials and Methods

When it comes to the measures of constructs, different sources were used for questionnaire formulation: attitudes and financial incentives were measured according to Baeli et al. [10], subjective norms were in accordance with Irawan et al. [31], and perceived behavioral control and e-bike purchase intention was similar to Li et al. [37]. Hereby, when it comes to respondents’ attitudes, after the introductory part of the formulation “Using an e-bike is for me”, nine different options were valuated. All the items (Table 2) were measured on a five-point Likert scale (from “strongly disagree” to “strongly agree”).

The research was conducted in 2022, relying on a convenience sample of students from the University of Novi Sad (Republic of Serbia). There were 332 respondents, of whom 55.7% were female. On average, respondents were 21.73 years old.

As for the sample size, we relied on Hair et al. [44], whose authors presented the approach based on the minimum R^2^ value. Hereby, for achieving an R^2^ value of 0.25 in the case of e-bike purchase intention (predicted by four variables), for a significance level of 5%, the minimum sample size should be 41. In addition to being larger than this threshold, our sample also exceeded the “10 times rule”, according to which “the minimum sample size should be 10 times the maximum number of arrowheads pointing at a latent variable anywhere in the PLS path model” [44] (p. 47), which is 40.

**Figure 2:**
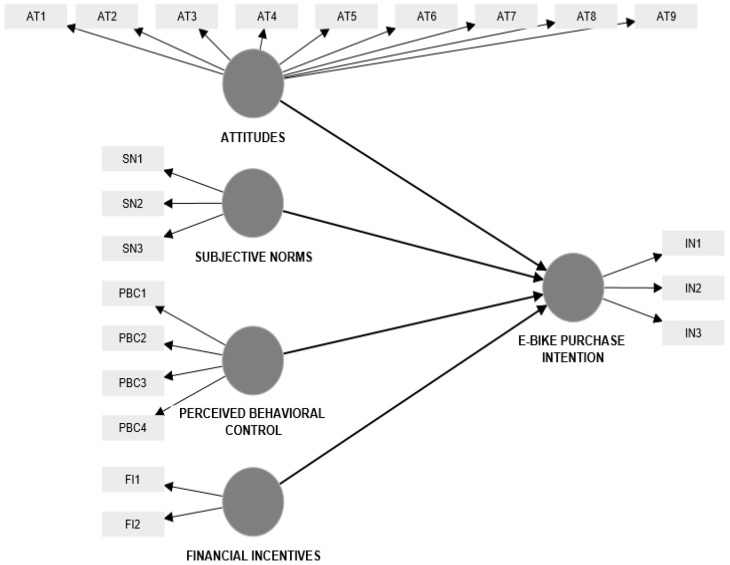
Statistical model—e-bike purchase intention.

The statistical model consisted of five first-order reflective constructs (Figure 2).

During data processing, partial least squares structural equation modeling (PLS-SEM) analysis [44] was implemented. Hereby, several steps were followed. Firstly, the examination of indicator loadings was implemented (a value greater than 0.70 is recommended for proving the reliability of each of the items). The assessment of internal consistency reliability by the use of Cronbach’s α and composite reliability (CR) (whereby values should be greater than 0.70) was then conducted. The assessment of convergent validity by the use of the average variance extracted (AVE) (for which values greater than 0.50 are acceptable) was performed next. Additionally, the assessment of discriminant validity by the use of the heterotrait–monotrait (HTMT) ratio of the correlations (where HTMT less than 0.90 can be acceptable) was implemented. The evaluation of the structural model included the analyses of collinearity and R^2^.

For testing all four hypotheses, we examined path coefficients between e-bike purchase intention and its predictors.

## 5. Results

### 5.1. Measurement Model

Having in mind that a value greater than 0.70 is recommended for proving the reliability of each of the items, all items with lower outer loadings (AT3, AT4, AT5, AT8, AT9, PBC1, and PBC4) were removed from further analysis. Table 3 presents the quality criteria of reflective constructs (outer loadings of remaining items, AVE, Cronbach’s α, and CR).

All outer loadings of the remaining items were greater than 0.70, confirming individual indicator reliability. Additionally, internal consistency reliability and discriminant validity were also confirmed; the values of Cronbach’s α and CR were greater than 0.70, while the values of AVE were greater than 0.50.

Table 4 presents the results of testing discriminant validity.

The values of HTMT were less than a threshold of 0.90 for all pairs, suggesting satisfactory results in terms of discriminant validity.

### 5.2. Structural Model

Having in mind that inner VIF values were less than 5, it can be concluded that there were no multicollinearity issues. The R^2^ value in the case of e-bike purchase intention was 0.743.

The results of the model, i.e., testing hypotheses, are presented in Table 5. It can be seen that the strongest positive and significant influence is related to financial incentives, thus confirming H4. It is followed by two significant, positive, and, from the strength aspect, relatively similar influences—those that can be attributed to attitudes and subjective norms, thus confirming H1 and H3, respectively. Finally, there was no significant influence of perceived behavioral control. Thus, H2 is rejected.

## 6. Discussion

The results of the research show that the strongest predictor of the intention to use e-bikes can be attributed to financial incentives. In addition, this intention is positively and significantly influenced by attitudes and subjective norms. The levels of influence of these two variables are relatively similar and far behind the influence of financial incentives. Finally, perceived behavioral control does not affect e-bike purchase intention significantly.

When considering the results of the research in the context of previous studies, several remarks should be added; hereby, it should be noted that our findings refer to the population of university students. The first is regarding the novelty of the research, namely, none of the cited studies included both TPB variables and financial incentives when considering e-bike choices. Generally, the volume of research dealing with e-bikes is rather limited and, as already suggested, there is a focus either on the context of TPB [35,36,37] or financial incentives [40,41,42]. There is only one study that includes both aspects, but it is related to conventional cycling [10]. Additionally, different research that implemented TPB regarding different aspects of cycling (intention to use, sharing, etc.), including e-cycling, relied on different variables. Hereby, the authors usually add variables to the original TPB model. Therefore, all the comparisons provided within this section should be treated with a certain level of caution.

The strongest influence of financial incentives on e-bike usage intention resembles results from previous research applying both TPB variables and financial incentives when considering conventional cycling in Italy [10]. However, the level of influence of attitudes in that research is similar to the one of financial incentives, while the influence of subjective norms and perceived behavioral control is less important. Nevertheless, the recommendation that financial incentives are substantial in promoting e-bike usage remains. It is also in accordance with previous research regarding e-cycling and financial incentives [40,41,42]. Therefore, if the authorities want to support such behavior among the student population, it would be of the greatest importance to provide adequate financial incentives, which is a known practice in different parts of the world, as presented in this manuscript.

When comparing the relative strength of different TPB predictors from this research with parallel results by other authors, several insights can be emphasized. Firstly, the strongest influence of attitudes, as is in this research, was also recorded in previous studies in Italy, Croatia, and China [10,30,32]. One of the research papers included, out of all standard TPB elements, only attitudes and found them important for e-cycling [36]. Additionally, the positive and significant influence of attitudes was also noticed in other studies [29,33,34,35,37]. There was only one research study, from Indonesia, where attitudes were not found to be significant [31].

Secondly, subjective norms being a positive and significant predictor of intention was also noticed in other studies [29,30,31,32,33,34,35,37]. However, while in most research regarding conventional cycling subjective norms is the least important predictor [29,30,31,32], when it comes to studies on e-cycling, it is the most important [35,37].

The results regarding attitudes and subjective norms from this research suggest that if students perceive that using an e-bike for each of them personally is easy, simple, exciting, and relaxing, their intention to use e-bikes would increase. The role of the media can be of special importance in strengthening such attitudes. In addition, if students perceive that people important to them, their student colleagues, or public opinion support such behavior, their intention would also rise. Advertising such support, especially on social media extensively used by this population, could also lead to a change in accordance with the desired behavior.

Thirdly, when it comes to perceived behavioral control, its lack of significance in this research is contrary to several previous studies [29,30,31,32,33,34,35,36], especially those in which that predictor is the most important [29,31,33,34]. Nevertheless, it is in accordance with one previous study regarding the physical activity of the student population from the same university in Serbia [45]. In that research, of different beliefs (behavioral, normative, and control) that correspond to elements of the theory of planned behavior (attitudes, subjective norms, and perceived behavioral control, respectively), behavioral and normative beliefs influenced intention positively and significantly, while control beliefs did not affect that variable. A possible explanation in the context of this research can arise from the consideration that perceived behavioral control was related to students’ knowledge of riding e-bicycles or being able to pay for them. The reason for the non-significant effect of PBC on intention could lie in the lower reliability of its measure (compared to other predictors), which might have decreased its prediction power [46]. Moreover, as found by Passafaro et al. [46], when it comes to university students, the strongest motivating factors usually refer to personal attitudes and social norms. Therefore, the strong effects of attitudes and social norms may attenuate the influence of perceived behavioral control [47].

## 7. Conclusions

The research presented within this manuscript had several starting points. Firstly, it considered the importance of physical activity for both the physical and psychological well-being of humans. Secondly, it directed the focus to the student population, bearing in mind the challenges associated with it when it comes to their physical activity. Thirdly, it was related to e-bikes because of their positive influence on riders’ physical activity and health, as well as on the environment.

Having previously described the intended framework, the authors included variables from the theory of planned behavior on one side and financial incentives on the other. Such a combination has already been used in previous research regarding cycling but, according to the authors’ knowledge, this is the first time that it has been implemented regarding e-bicycles. E-cycling was previously considered from only one of these two aspects, thus not providing the possibility to compare their relative influence on choosing e-bikes. In addition, similar research is scarce in the context of the country where the research was conducted.

The results highlight the greater importance of financial incentives in comparison to variables from TPB from the aspect of the intention to purchase e-bikes. The implications are important for the authorities, suggesting the need to offer such incentives for increasing the use of e-bikes. Nevertheless, two out of three variables from the theory of planned behavior also proved to be influential. Therefore, there is a need to strengthen young people’s positive attitudes towards e-bikes, as well as the social pressure to use them. The role of the media can be emphasized from that aspect.

The authors believe that this research provides both theoretical and practical implications. Future research can focus on the wider population and respondents from different parts of the country, as well as on additional aspects of green transport and/or physical activity.

## Figures and Tables

**Table 1:** Incentives on a national level for pedelec-25 [43].

Country	Target Group	Incentives
Finland	Individuals	EUR 1000
France		EUR 400
Hungary		HUF 150,000
Lithuania		EUR 700
Malta		EUR 250
Croatia	Business/Associations	HKR 4995
Greece	Business + Individuals	EUR 800
Portugal		EUR 500
Norway	Public entities	NOK 15,000
Ireland	All groups	Tax incentive

**Table 2:** Measurement items.

Constructs and Items
**Attitudes** “Using an e-bike is for me—easy (AT1),—simple (AT2),—cheap (AT3),—safe (AT4), —comfortable (AT5),—exciting (AT6),—relaxing (AT7),—fast (AT8),—useful (AT9)”
**Subjective norms**
“Most people who are important to me think that I should use an e-bike” (SN1) “My student colleagues support me to use an e-bike” (SN2) “Public opinion affects my choice to use an e-bike” (SN3)
**Perceived behavior control**
“I have the skills to ride e-bikes” (PBC1) “I have the knowledge to use e-bikes” (PBC2) “I’m able to pay for an e-bike” (PBC3) “I have the psychological qualities to deal with riding risks” (PBC4)
**Financial incentives** “Authorities’ incentives for purchasing an e-bike encourage me to buy it” (FI1) “I think I will use the authorities’ incentives for purchasing e-bikes” (FI2)
**E-bike purchase intention** “I will try to purchase an e-bike” (IN1) “I will recommend others to purchase e-bikes” (IN2) “I intend to purchase an e-bike as a feasible way to travel in the future” (IN3)

**Table 3:** Quality criteria.

Constructs and Items	Loadings	AVE	CR	Cronbach’s α
**Attitudes**		0.735	0.917	0.881
AT1	0.895			
AT2	0.845			
AT6	0.814			
AT7	0.874			
**Subjective norms**		0.729	0.890	0.818
SN1	0.880			
SN2	0.905			
SN3	0.771			
**Perceived behavioral control**		0.781	0.877	0.719
PBC2	0.877			
PBC3	0.890			
**Financial incentives**		0.909	0.952	0.900
FI1	0.953			
FI2	0.954			
**Purchase intention**		0.882	0.957	0.933
IN1	0.958			
IN2	0.917			
IN3	0.941			

**Table 4:** HTMT approach—discriminant validity.

	HTMT
FI → AT	0.537
IN → AT	0.672
IN → FI	0.878
PBC → AT	0.184
PBC → FI	0.195
PBC → IN	0.193
SN → AT	0.607
SN → FI	0.391
SN → IN	0.572
SN → PBC	0.150

**Table 5:** Path coefficients.

Path Coefficients	Direct Effect	*p*-Value	Hypotheses
AT → IN	0.212	0.000	H1 Accepted
PBC → IN	0.006	0.844	H2 Rejected
SN → IN	0.191	0.000	H3 Accepted
FI → IN	0.635	0.000	H4 Accepted

## Data Availability

Not applicable.
